# The effects of Slavin model-based time management training on procrastination in nursing students: a quasi-experimental study

**DOI:** 10.3389/fpsyg.2026.1756887

**Published:** 2026-02-25

**Authors:** Seda Tugba Baykara Mat

**Affiliations:** Department of Nursing, Faculty of Health Sciences, Istanbul Beykent University, Istanbul, Türkiye

**Keywords:** procrastination, program evaluation, students, nursing, teaching, time management

## Abstract

**Introduction:**

Nursing students frequently experience time management difficulties as they attempt to balance intensive theoretical coursework with demanding clinical responsibilities. When time is not managed effectively, procrastination often develops, leading to academic strain, heightened stress, and reduced readiness for professional practice. This study aimed to examine the effectiveness of a theory-based time management training program grounded in Slavin’s Effective Teaching Model in reducing procrastination and improving time management skills among nursing students.

**Methods:**

A quasi-experimental pretest–posttest design was used with 62 undergraduate nursing students enrolled at a single university. The intervention consisted of a structured 10-hour time management training program delivered over multiple sessions. The program emphasized practical, evidence-based strategies such as planning, prioritizing tasks, creating personalized schedules, and applying time-blocking techniques in daily academic life. Data were collected using the General Procrastination Tendency Scale and the Academic Procrastination Scale, with a particular focus on the Effective Use of Time subdimension. Because the data were not normally distributed, nonparametric statistical analyses were conducted.

**Results:**

Following the training program, students demonstrated a significant improvement in Effective Use of Time scores and a significant decrease in academic procrastination levels. These changes suggest that participants became more organized, managed their academic tasks more effectively, and engaged more consistently with their coursework. However, no significant change was observed in overall general procrastination tendencies.

**Discussion:**

The findings indicate that structured, theory-based time management training can effectively improve functional time management behaviors and reduce context-specific academic procrastination among nursing students. The absence of change in general procrastination suggests that more stable, trait-like procrastination patterns may require longer-term or more intensive interventions. Incorporating theoretically grounded time management training into nursing education may support academic self-regulation, enhance academic preparedness, and contribute to stronger clinical performance in demanding learning environments.

**Clinical trial registration:**

NCT06675838 (2024-11-04).

## Introduction

Procrastination, defined as the voluntary delay of intended tasks despite expecting negative consequences, is a pervasive self-regulation difficulty among university students (Steel, 2007; Khan et al., 2024). Nursing students, who must juggle demanding theoretical coursework and time-sensitive clinical responsibilities, are considered an at-risk group for maladaptive time management behaviors.

Recent empirical evidence indicates that the prevalence of procrastination among nursing students ranges from moderate to high. In a large multi-institutional study in Türkiye, 62.4% of nursing students demonstrated moderate levels of academic procrastination, and poor time management was a significant predictor (Çingöl and Karakaş, 2023). A study conducted in India similarly reported that 68% of nursing students exhibited moderate procrastination, with heavy academic workload and avoidance behaviors noted as contributing factors (Yousuf et al., 2024). In Indonesia, Khotimah et al. (2022) found a strong negative correlation between time management ability and academic procrastination among nursing students, strengthening the behavioral relevance of this pattern.

From a behavioral-health perspective, procrastination has broader implications beyond academic performance. The Procrastination–Health Model (Tice and Baumeister, 1997; Sirois et al., 2003; Sirois and Pychyl, 2013) posits two mechanisms linking procrastination to poorer well-being:

1. Stress Pathway – Delaying tasks increases psychological stress as deadlines approach, impairing emotional regulation.
2. Behavioral Pathway – Procrastination leads individuals to postpone health-promoting habits such as sleep, physical activity, and self-care behaviors, resulting in cumulative negative effects on health.

Empirical evidence supports these pathways. For example, Sirois (2023) demonstrated that chronic procrastination leads to poorer health outcomes by elevating stress levels and diminishing engagement in self-care behaviors. Likewise, Johansson et al. (2023) found that higher levels of procrastination correlate with poorer self-rated health and more frequent physical complaints.

In educational contexts, procrastination is understood as a failure of self-regulation, particularly in time management. Research demonstrates that well-structured time management interventions can reduce academic procrastination and improve students’ academic performance (Zhao et al., 2021). However, the effectiveness of such interventions depends on the pedagogical quality and structure of instruction.

In the post-pandemic higher education context, instructional delivery methods have diversified, with increasing interest in online and hybrid formats. Emerging evidence suggests that learning effectiveness and student engagement may be comparable across instructional modalities, highlighting the need for theory-driven and scalable educational interventions (So et al., 2025).

Slavin’s Effective Teaching Model (1984) offers a strong pedagogical framework that highlights the central role of instructional quality, the suitability of instruction to learners’ needs, motivational support, and active use of learning time. These elements closely align with the cognitive and behavioral skills essential to effective time management.

Given the high prevalence of procrastination among nursing students and the documented impact on well-being and academic success, there is a clear need for theory-driven, evidence-based time management interventions within nursing education. This study, therefore, aimed to evaluate the effectiveness of a structured time management training program—grounded in Slavin’s Effective Teaching Model and informed by the Procrastination–Health Model—in improving time use and reducing procrastination behaviors among nursing students.

By integrating an educational model with a behavioral-health model, this study contributes to nursing science through a dual-theoretical perspective on self-regulation and professional preparedness.

## Methods

### Research design

The single-group pretest–posttest design was selected as a pragmatic approach commonly used to evaluate educational interventions across cohorts and delivery formats. Consistent with recent large-scale program evaluations emphasizing participation, attendance, and completion as key implementation indicators (So et al., 2025), participant engagement and session attendance were systematically monitored to document intervention dose and fidelity.

### Theoretical framework

The time-management training program was grounded in Slavin’s Effective Teaching Model, which is conceptually derived from Carroll’s (1963) *Model of School Learning* and operationalized through the QAIT framework (Quality of instruction, Appropriateness, Incentive, and Time). According to this framework, effective learning is most likely to occur when instruction is delivered with high quality, aligned with learners’ prior knowledge and skill levels, supported by motivational incentives, and accompanied by sufficient and well-managed learning time.

The time-management training program was grounded in Slavin’s Effective Teaching Model, which is conceptually rooted in Carroll’s (1963) Model of School Learning and operationalized through the QAIT framework—Quality of instruction, Appropriateness, Incentive, and Time. Within this framework, effective learning is most likely to occur when instruction is delivered with high quality, tailored to learners’ existing knowledge and skill levels, supported by appropriate motivational incentives, and conducted within sufficient and well-structured learning time.

In addition to Slavin’s model, the intervention was informed by the Procrastination–Health Model (Tice and Baumeister, 1997; Sirois, 2023), which conceptualizes procrastination as a self-regulation failure linked to stress and maladaptive coping behaviors. This model provided a complementary behavioral lens for identifying intervention targets, particularly in relation to academic procrastination and time-use behaviors.

Together, these theoretical frameworks ensured that the training program was both pedagogically grounded and behaviorally focused. A systematic mapping of each training session to the relevant QAIT components was used to maintain theoretical coherence and intervention fidelity throughout the program.

### Variables

The independent variable was the structured time management training delivered to senior (intern) nursing students. The dependent variable was the level of procrastination behaviors exhibited by these students. The central hypothesis tested in the study was:

*H1*: Time management training provided to intern nursing students reduces their procrastination behaviors.

This hypothesis was evaluated by comparing pre-training and post-training measurements.

### Setting, participants, and sample adequacy

The study was conducted at a foundation university in Istanbul, Türkiye. All senior nursing students who were actively working as interns during the study period constituted the accessible population (*N* = 70), of whom 62 completed both pretest and posttest assessments and were included in the final analyses. Recruiting students from a single institution ensured consistency in exposure to the same curriculum and clinical training standards, thereby reducing variability related to educational background. Although nursing curricula across Türkiye share nationally defined core requirements, differences in instructional delivery, pedagogical emphasis, and clinical exposure hours across institutions may influence learning experiences; therefore, focusing on a single program enhanced internal consistency.

With a final sample size of *N* = 62 paired observations, a sensitivity power analysis was conducted using G*Power version 3.1.9.7 (Heinrich Heine University Düsseldorf, Germany). Assuming a matched-pairs design, a two-tailed significance level of *α* = 0.05, and a desired statistical power of 0.80, the analysis indicated that the study was powered to detect small-to-moderate and larger effects (minimum detectable effect size dz. ≈ 0.36). For this conservative approximation of paired nonparametric tests, a moderate correlation between repeated measures (r = 0.50) was assumed. These findings support the adequacy of the sample size for detecting meaningful pre–post changes in educational intervention outcomes.

### Intervention procedures

The time management training was designed as an evidence-based, structured program focusing on goal setting, prioritization, scheduling, and task planning, aligned with self-regulation principles. The training was delivered face-to-face in the classroom over a series of structured sessions.

#### Development of the training content

The final version includes the following:

1) Source materials

The training content was developed through an extensive literature review encompassing time management interventions in nursing education, procrastination and self-regulation theories, Slavin’s Effective Teaching Model, and the behavioral pathways outlined in the Procrastination–Health Model. Key sources informing this process included Çingöl and Karakaş (2023), Zhao et al. (2021), Khotimah et al. (2022), and Sirois (2023).

2) Expert review

A three-member expert panel evaluated the training manual, consisting of two Assistant Professors specializing in nursing education and one faculty member in educational sciences. The experts reviewed the manual for content validity, theoretical alignment, clarity of objectives, and the suitability of examples for nursing students. Revisions were incorporated based on their feedback. Following these expert-driven modifications, the session objectives, instructional strategies, and evaluation criteria were finalized. Each session was explicitly designed to address one or more components of the QAIT framework to ensure theoretical coherence and instructional fidelity. The finalized details are presented in Table 1.

**Table 1:** Time management training program aligned with the QAIT framework.

Session (Day and Duration)	Educational focus	Instructional strategies	QAIT component(s)	Expected learning outcomes
Day 1–2 h	Introduction to time management and procrastination	Lectures, structured informational materials	Quality, appropriateness	Increased awareness of time management concepts and recognition of procrastination patterns
Day 2–3 h	Time management strategies and planning techniques	Interactive activities, applied planning exercises	Quality, appropriateness, time	Improved prioritization, scheduling, and allocation of academic time
Day 3–2 h	Understanding and addressing procrastination	Self-reflection tasks, guided group discussions	Appropriateness, incentive	Enhanced insight into personal procrastination triggers and motivation to change
Day 4–3 h	Hands-on application and time planning workshop	Group tasks, real-life academic simulations	Quality, time	Development of individualized and practical time-management strategies
Day 5–2 h	Evaluation, feedback, and motivational reinforcement	Peer evaluation, feedback sessions, motivational incentives	Incentive, time	Strengthened self-monitoring skills and reinforcement of sustained time-management behaviors

### Intervention

This educational program was developed to enhance nursing students’ time management skills, reduce procrastination behaviors, and support academic performance, with the broader goal of fostering professional success after graduation. The instructional design was informed by Slavin’s QAIT model (Quality, Appropriateness, Incentive, and Time), which builds on Carroll’s (1963) Model of School Learning and highlights the importance of time-on-task in achieving meaningful learning outcomes.

Within the QAIT framework, learning is optimized by addressing four interrelated elements: high-quality instruction, alignment of instructional level with learners’ readiness, meaningful motivational incentives, and sufficient opportunities for learning over time (Slavin, 2018). Owing to its emphasis on structured yet flexible group instruction, this model has been widely used in educational intervention research. In the present study, the time-management training program was intentionally designed to incorporate each QAIT component, with particular attention given to effective use of learning time and motivational strategies aimed at reducing academic procrastination.

The first component, instructional quality, emphasizes the delivery of content that is engaging, meaningful, memorable, and applicable. Structuring knowledge coherently, linking new concepts to prior learning, and integrating visual or multimedia tools enhance comprehension and retention. High-quality instruction involves clear presentation, relevant examples, and timely feedback to facilitate knowledge transfer. The second component, appropriate levels of instruction, addresses the alignment of course content with learners’ abilities, attitudes, learning speeds, and motivational levels. While full individualization may not be feasible in group settings, adopting flexible strategies ensures that individual differences are acknowledged and addressed effectively. The third component, incentive, relates to motivating students to engage actively with the learning process and internalize content. Demonstrating the real-life and professional relevance of acquired knowledge can significantly boost intrinsic motivation and attentiveness. The fourth component, time, is conceptualized as a contextual factor whose effectiveness depends on instructional quality, appropriateness, and motivation. The model distinguishes between allocated time (designated by the instructor or institution) and engaged time (actual time students are actively involved in learning). Simply increasing instructional time does not guarantee better learning; rather, it should be used efficiently to maximize engagement and comprehension. Attendance records showed that all participants completed the entire training program.

By integrating these components, the program was implemented as part of the “Management and Organization” course during the final academic term. The training aimed to instill effective time management practices, reduce procrastination tendencies, and promote structured academic engagement, thereby preparing nursing students for both academic and professional demands.

Table 2 provides a concise summary of the time management training program implemented for nursing students. Each session is aligned with the educational focus, key instructional strategies, and anticipated learning outcomes. This format supports clarity and coherence within the manuscript’s methodology and intervention sections.

**Table 2:** Training goals, objectives, and strategies.

Training goal	Related objectives	Relevant content and strategies
1. Enhancing time management awareness	Teach the importance and strategies of time management Emphasize its impact on academic and professional life	Definition and importance of time management Principles: prioritization, time blocking, planning
2. Reducing procrastination	Help students recognize procrastination habits • Understand causes and develop coping strategies	Definition, causes, and impact of procrastination Self-reflection and behavior management
3. Teaching practical time management strategies	Provide practical techniques applicable in daily life Teach prioritization, time blocking, and planning	Task Prioritization Time Blocking Daily Planning Creating and applying personal time plans

### Data collection

Data were collected in person immediately before and after the training using a Demographic Information Form and a validated measure of procrastination. Both pre-test and post-test surveys were administered under the supervision of the research team to ensure completeness and accuracy of responses. The inclusion criteria were: (1) being over the age of 18; (2) being officially enrolled as an intern nursing student at the university where the study was conducted; (3) actively attending formal education; (4) possessing adequate proficiency in understanding and speaking Turkish; and (5) voluntarily agreeing to participate in the research.

The exclusion criteria were: (1) transferring to another department or institution during the study period, and (2) withdrawing from the study at any stage. Posttest data were collected immediately after completion of the final session.

### Intervention fidelity and participant flow

Participant attendance was monitored throughout the intervention using session-specific attendance records. The time-management training program consisted of five sessions. Most participants attended all sessions, and no participant missed more than one session. When a session was missed, participants received a brief summary of the session content to ensure continuity of the intervention. Intervention fidelity was supported through standardized session plans, instructor checklists, and participant worksheets aligned with the predefined learning objectives and activities of each session.

A total of 70 undergraduate nursing students were assessed for eligibility. Of these, 62 completed the intervention and both pre-test and post-test assessments and were included in the final analyses. Participants who did not complete the study were excluded due to absence from training sessions or incomplete post-test data. Analyses were conducted using complete-case data; therefore, no imputation methods were applied for missing values.

### Data collection tools

#### Student information form

This form was developed by the researchers and consists of 13 items aimed at collecting participants’ socio-demographic characteristics and daily time management habits. The first six questions elicit demographic data such as gender, age, and parents’ education and occupation. The remaining seven questions assess students’ daily planning skills, approaches to time use, and attitudes toward structured routines.

#### General procrastination scale

The General Procrastination Scale was developed by Çakıcı (2003) to measure general procrastination tendencies among students 0.25. This instrument consists of 18 items evaluated on a 5-point Likert scale (1 = “Does not describe me at all” to 5 = “Describes me completely”) and comprises two subscales: Procrastination (items 2, 5, 7, 9, 10, 11, 13, 14, 15, 16, 18) and Effective Time Management (items 1, 3, 4, 6, 8, 12, 17). In Çakıcı’s original study, the Cronbach alpha coefficient for the Procrastination subscale was 0.88 and for the Effective Time Management subscale was 0.85. In this study, the Cronbach alpha coefficient for the general scale is 0.830, indicating high internal consistency. Higher total scores in the Procrastination subscale and on the overall scale indicate greater general procrastination tendencies. Conversely, higher scores in the Effective Use of Time subscale reflect more effective planning, organization, and time management behaviors. Subscale scores were calculated by summing the item responses within each dimension, and the total score was obtained by summing all items.

#### Academic procrastination scale

The Academic Procrastination Scale, developed by Çakıcı (2003), is used to assess individuals’ tendency to delay academic tasks. The scale consists of 19 items rated on a 5-point Likert scale, including 12 negatively worded items and 7 positively worded items (Items 1, 4, 7, 9, 11, 13, and 17). The positively worded items were reverse-coded prior to analysis to ensure consistent scoring. After reverse coding, higher total scores indicate a greater tendency toward academic procrastination. Total scores were calculated by summing responses to all items. The original reliability coefficient of the scale was reported as 0.92. In the present study, the Cronbach’s alpha coefficient was 0.94, indicating excellent internal consistency.

### Data analysis

All statistical analyses were conducted using IBM SPSS Statistics version 26.0 (IBM Corp., Armonk, NY, USA). Descriptive statistics, including mean, standard deviation, median, minimum, maximum, frequency, and percentage, were used to summarize participants’ socio-demographic characteristics and responses to time-management–related items.

Prior to inferential analyses, the normality of pre–post difference scores (post-test minus pre-test) for each scale and subscale was assessed using the Shapiro–Wilk test, which is recommended for small to moderate sample sizes. The results indicated that the difference scores for the General Procrastination Tendency Scale and the Procrastination subdimension deviated from normality, whereas the Time Management subdimension and the Academic Procrastination Scale did not fully violate normality assumptions. Given the mixed distributional properties of the outcome variables and the modest sample size, a nonparametric analytical approach was adopted to ensure consistency and statistical robustness across analyses.

Accordingly, the Wilcoxon signed-rank test was used to compare pre-test and post-test scores as a nonparametric alternative to the paired-samples *t*-test. Effect sizes were calculated using the formula *r* = Z/√N, which is appropriate for Wilcoxon signed-rank tests. Effect size values were interpreted as 0.10 = small, 0.30 = moderate, and 0.50 = large. All statistical tests were two-tailed, and statistical significance was set at *p* < 0.05. Only participants with complete pre-test and post-test data were included in the analyses; therefore, missing data were handled using a complete-case approach.

## Results

Although the change in the total score of the General Procrastination Tendency Scale was not statistically significant, the slight reduction in mean values may indicate an early cognitive shift rather than an immediate behavioral change. In contrast, a statistically significant increase was observed in the *Effective Use of Time* subscale, suggesting a meaningful improvement in students’ planning, prioritization, and scheduling behaviors. Additionally, a significant decrease in Academic Procrastination Scale scores was found following the intervention, indicating a reduction in academic procrastination behaviors. These findings suggest that the Slavin model–based time management training was effective in enhancing time management skills and reducing academic procrastination, even though broader procrastination tendencies may require longer-term interventions to demonstrate substantial change.

**Figure 1:**
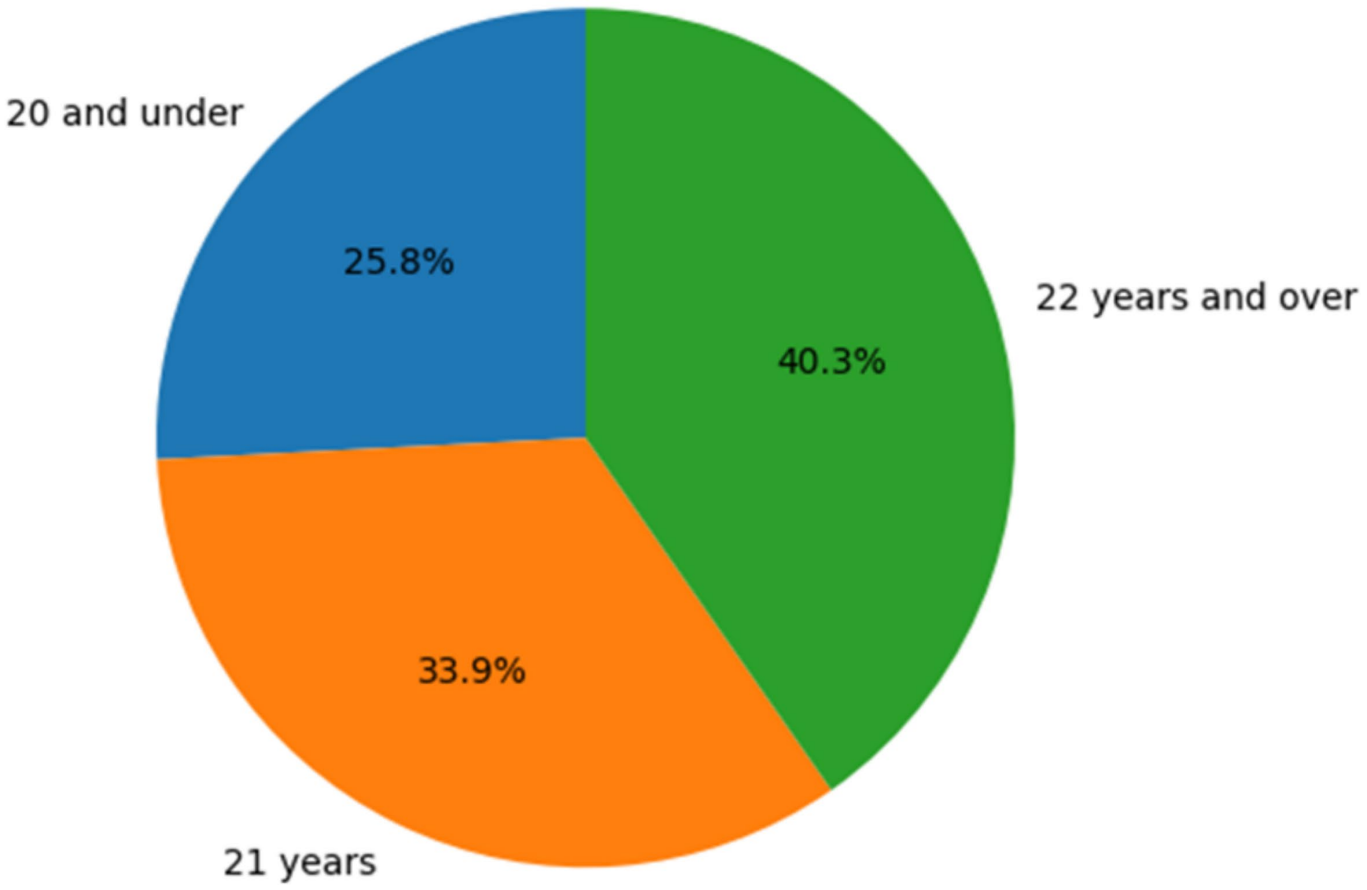
Distribution of participants by age group presents here.

**Figure 2:**
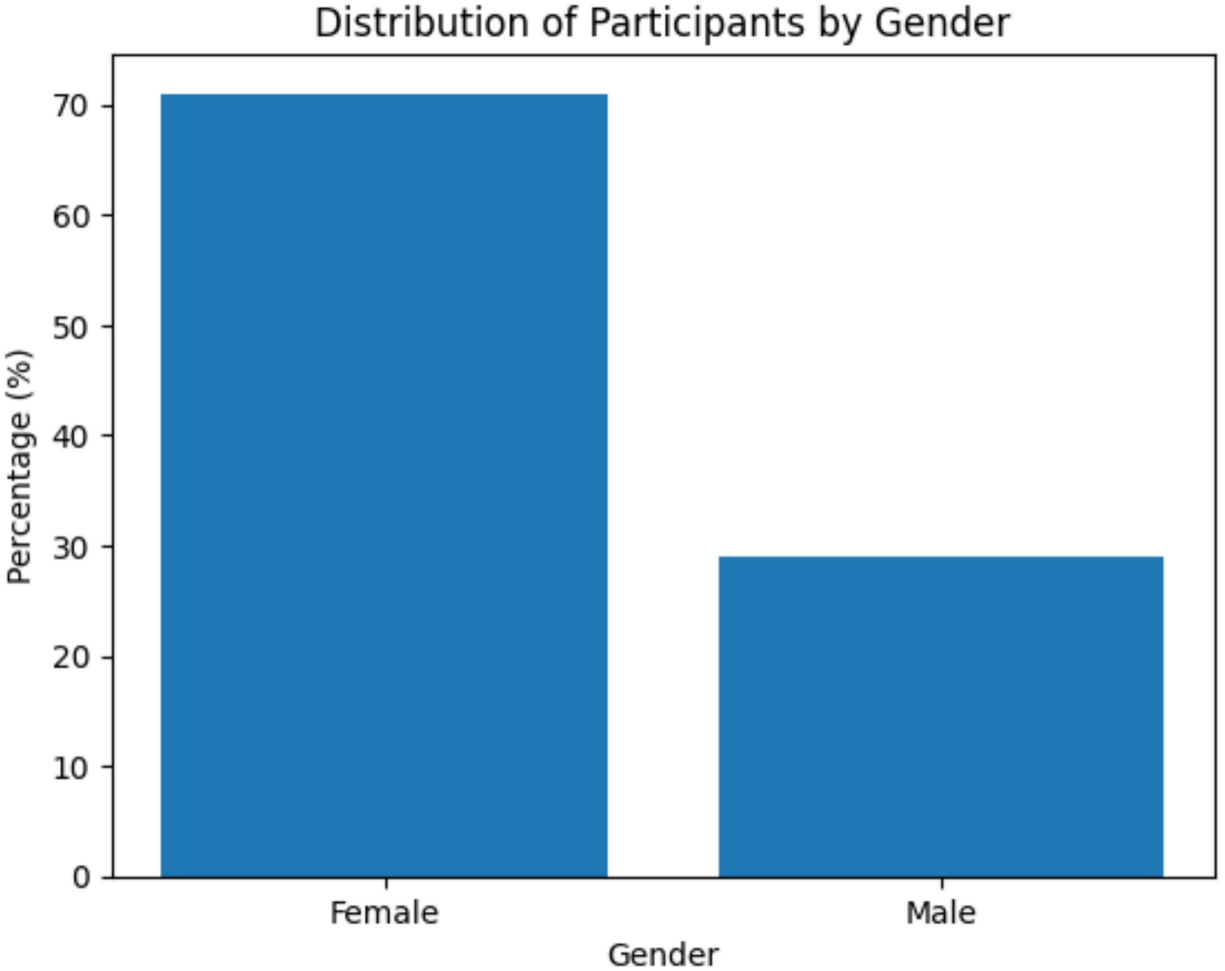
Distribution of participants by gender presents here.

As shown in Figures 1, 2, the largest proportion of participants were aged 22 years and over (40.3%), followed by those aged 21 years (33.9%) and 20 years and under (25.8%). In terms of gender distribution, the majority of participants were female (71.0%), while males accounted for 29.0% of the sample.

**Figure 3:**
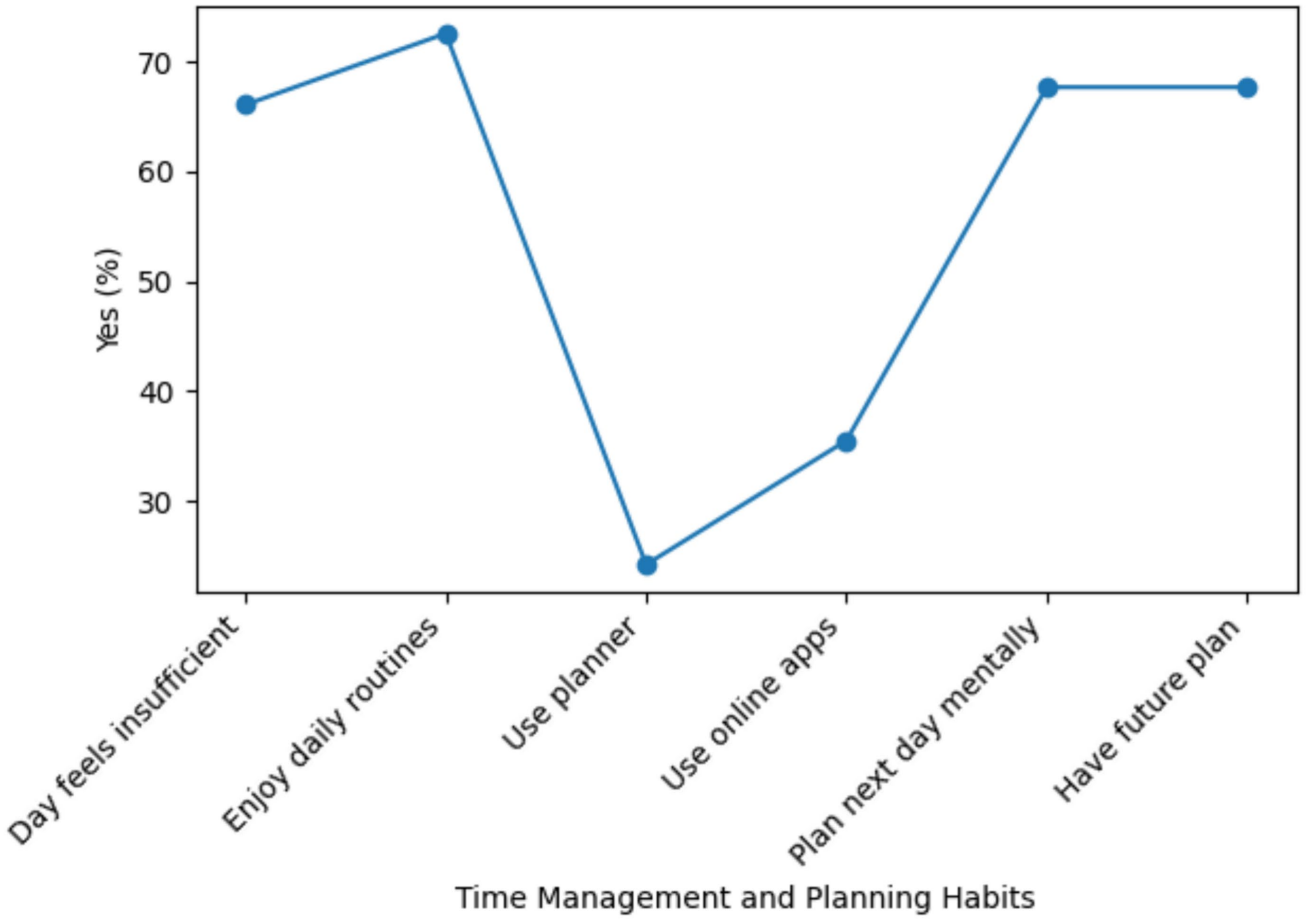
Time management and planning habits of participants present here.

As shown in Figure 3, 66.1% of the participants reported that a single day was generally insufficient to complete their tasks, while 72.6% stated that they enjoyed their daily routines. Despite this, a large proportion of students reported not using structured planning tools, with 75.8% indicating that they did not use an agenda and 64.5% reporting that they did not utilize online applications to organize their daily activities. In contrast, 67.7% of the participants reported mentally planning the following day, and the same proportion indicated having a mental plan regarding their future. Overall, these findings suggest that while nursing students tend to rely on cognitive and informal planning strategies, the use of formal time-management tools remains limited, underscoring the need for structured time-management training within nursing education programs.

As presented in Table 3, the pre-test mean score of the General Procrastination Tendency Scale was 50.53 ± 12.51, while the post-test mean score slightly decreased to 50.00 ± 8.03. Similarly, the mean score of the Procrastination subscale decreased from 26.21 ± 12.38 at pre-test to 23.65 ± 7.71 at post-test. In contrast, the Effective Use of Time subdimension showed an increase in mean scores, rising from 24.32 ± 6.64 before the intervention to 26.35 ± 4.36 after the intervention. Notably, the Academic Procrastination Scale mean score demonstrated a substantial decrease from 54.11 ± 13.10 at pre-test to 44.88 ± 10.40 at post-test, indicating a reduction in academic procrastination following the intervention.

**Table 3:** Descriptive statistics of the scales used in the study.

Scales	Mean	SD	Median	Min.	Max.
General procrastination tendency scale – total (Pre)	50.53	12.51	48.00	21.00	84.00
Procrastination subscale (Pre)	26.21	12.38	23.50	11.00	54.00
Time management subscale (Pre)	24.32	6.64	25.00	8.00	35.00
Academic procrastination scale (Pre)	54.11	13.10	54.50	27.00	86.00
General procrastination tendency scale – total (Post)	50.00	8.03	49.00	41.00	72.00
Procrastination subdimension (Post)	23.65	7.71	22.00	12.00	43.00
Effective use of time subdimension (Post)	26.35	4.36	27.50	16.00	34.00
Academic procrastination scale (Post)	44.88	10.40	42.50	27.00	63.00

Mean: Arithmetic Mean – SD: Standard Deviation – Pre: Pre-test – Post: Post-test.

As shown in Table 4, no statistically significant differences were observed between pre-test and post-test scores for the total score of the General Procrastination Tendency Scale (*Z* = −0.15, *p* = 0.877) or for its Procrastination subscale (*Z* = −1.46, *p* = 0.142). These findings suggest that general procrastination tendencies remained relatively stable over the course of the intervention.

**Table 4:** Pretest–posttest comparison of procrastination and time-management subscales.

Scales	Variables	*n*	Mean	SD	MR	Analysis results	ES*	95% Confidence interval (CI)
General procrastination tendency scale – total score	Pre-test	62	50.53	12.50	29.21	Z: –0.155 *p*: 0.877	---	---
	Post-test		50.00	8.03	30.88			
Procrastination subscale	Pre-test	62	26.20	12.38	33.77	Z: –1.46 p: 0.142	---	---
	Post-test		23.64	7.71	26.50			
Time management subscale (Pre)	Pre-test	62	24.32	6.63	29.86	Z:–2.29 *p*: 0.022	0.36*	[−0.50,−0.08]
	Post-test		26.35	4.35	28.60			
Academic procrastination scale	Pre-test	62	54.11	13.10	36.96	Z:–3.78 *p*: 0.000	0.77*	[−0.65, −0.26]
	Post-test		44.88	10.40	20.83			

Wilcoxon signed-rank tests were used for all analyses. Effect sizes were calculated using the formula *r* = Z/√N. The following benchmarks were applied for interpretation of r: 0.10 = small, 0.30 = medium, and 0.50 = large effect. Negative *r* values indicate lower post-test scores compared with pre-test scores. Confidence intervals are reported at the 95% level.

In contrast, a statistically significant improvement was observed in the Effective Use of Time subscale, with post-test scores higher than pre-test scores (*Z* = −2.29, *p* = 0.022). The corresponding effect size indicated a small-to-moderate magnitude of change (*r* = 0.29), suggesting a meaningful, albeit modest, improvement in students’ time-management behaviors following the training.

Additionally, scores on the Academic Procrastination Scale showed a statistically significant decrease after the intervention (*Z* = −3.78, *p* < 0.001). The associated effect size was in the medium-to-large range (*r* = 0.48), indicating a notable reduction in academic procrastination. Taken together, these results suggest that while broader procrastination tendencies may be relatively resistant to short-term change, the intervention was associated with meaningful improvements in context-specific academic procrastination and functional time use.

## Discussion

The present study demonstrated that a time-management training program grounded in Slavin’s Effective Teaching Model was associated with a significant reduction in nursing students’ academic procrastination and a significant improvement in their effective use of time. These findings suggest that students became more capable of planning, prioritizing, and structuring their academic responsibilities—skills consistently linked to academic performance and self-regulated learning in nursing education (Çingöl and Karakaş, 2023; Zhao et al., 2021). The results align with earlier evidence, including Britton and Tesser’s (1991) foundational work, indicating that structured, theory-informed instruction in planning and scheduling can strengthen students’ time-management behaviors and academic engagement. In line with more recent studies, our findings also suggest that self-regulation interventions embedded within a coherent pedagogical framework may be particularly effective in addressing context-specific academic procrastination (Khotimah et al., 2022; Suárez Perdomo and Ruiz Alfonso, 2024). Importantly, higher scores on the Effective Use of Time subdimension indicate more effective time-management behaviors; therefore, the observed post-test increase reflects an improvement in students’ functional planning and scheduling skills rather than an increase in procrastination.

Despite these improvements, no statistically significant changes were observed in the total score of the General Procrastination Tendency Scale or its Procrastination subdimension. This finding suggests that deeply ingrained procrastination tendencies may be less responsive to short-term, skills-focused interventions and may instead require longer-term or multi-component approaches to change. This interpretation is consistent with previous research characterizing general procrastination as a relatively stable, trait-like pattern that is resistant to brief educational programs (Sirois, 2023; Johansson et al., 2023). From the perspective of the Procrastination–Health Model (Tice and Baumeister, 1997; Sirois, 2023; Sirois and Pychyl, 2013), the present findings may reflect a distinction between early cognitive and motivational shifts triggered by the intervention and the slower translation of these changes into broader, habitual behavior. Accordingly, the observed reduction in academic procrastination may represent an initial stage of change, whereas the stability of general procrastination scores suggests that sustained practice, reinforcement, or extended intervention duration may be necessary for more pervasive behavioral change to occur.

Following clarification of the Academic Procrastination Scale scoring, the observed decrease in APS scores indicates a meaningful reduction in academic procrastination after the intervention. This improvement can be directly linked to the active components of the training program, particularly the structured planning exercises and the time-blocking workshop, which encouraged students to allocate study time more deliberately and engage with academic tasks in a timely manner. Within Slavin’s QAIT framework, these components primarily address the “Time” dimension by increasing engaged learning time and the “Appropriateness” dimension by aligning strategies with students’ academic demands and skill levels. Additionally, goal-setting, feedback, and self-monitoring activities likely contributed through the “Incentive” component by enhancing motivation and sustained task engagement, while the structured and interactive delivery of content supported instructional “Quality.” Together, these elements provide a coherent pedagogical explanation for the observed reduction in academic procrastination.

Several elements of Slavin’s Effective Teaching Model (1984) likely contributed to the effectiveness of the intervention. High-quality instruction—including clear examples, planning templates, and structured learning tasks—may have enhanced comprehension and supported skill acquisition, consistent with recent work on instructional quality in nursing education (Slavin, 1984). Instructional appropriateness, tailored to the unique academic and clinical demands of nursing students, likely promoted engagement and self-regulation (Çingöl and Karakaş, 2023). Likewise, the incentive component, reinforced through motivational feedback and explicit links between time management and future professional roles, may have strengthened students’ intrinsic motivation, paralleling evidence that motivational processes shape procrastination behaviors (Eren and Coşkun, 2016). Active learning strategies, including time-blocking workshops and weekly planning exercises, also appear to have facilitated the translation of instruction into practice, as reflected by improvements in the Effective Use of Time subscale. Taken together, these mechanisms support the notion that Slavin’s instructional framework can enhance academic self-regulation among nursing students.

From an educational perspective, the findings underscore the value of integrating structured time-management training into nursing curricula. Given the intensity of both academic and clinical demands in nursing education, supporting students in developing effective time-use habits early in their training may be critical for academic success and professional preparedness (Zhao et al., 2021). While the current intervention demonstrated positive effects on academic procrastination and functional time use, future programs may benefit from incorporating additional components such as cognitive–behavioral strategies, stress-management techniques, and self-efficacy enhancement to more comprehensively address the behavioral and motivational dimensions of procrastination (Rabin et al., 2011; Salguero-Pazos and Reyes-de-Cózar, 2023). Moreover, longitudinal research is needed to examine whether these improvements are sustained over time and to explore potential moderating factors, such as individual characteristics and demographic variables, that may influence the effectiveness of the intervention. Although a separate manipulation check was not conducted, the Effective Use of Time subdimension served as a meaningful indicator of participants’ engagement with the intervention, as it directly captures the planning and scheduling behaviors targeted by the training. Improvements observed in this subdimension therefore suggest that participants actively applied the strategies introduced during the program, providing indirect support for the effectiveness of the intervention (Sirois et al., 2003).

Beyond the observed outcomes within a face-to-face instructional format, the present findings also point to broader implications for scalability and instructional delivery. Recent multi-year evidence from higher education indicates that learning effectiveness, student participation, and satisfaction may be largely comparable across online and face-to-face instruction. In a large pre–post analysis spanning five academic years, So et al. (2025) reported similar levels of enrollment, attendance, and satisfaction across 118 face-to-face and 110 online courses, supporting the equivalence of instructional modalities and highlighting the potential of hybrid delivery models.

In this context, the theory-driven time-management training evaluated in the present study appears well suited for adaptation to online or hybrid formats without compromising core educational outcomes. Consistent with QAIT principles, such adaptations could maintain instructional quality, appropriateness, incentive, and engaged learning time while expanding access and reach. Future implementations may also benefit from aligning outcome monitoring with participation indicators emphasized by So et al. (2025), including enrollment, attendance, completion, and satisfaction.

Because the study was conducted within a single nursing program, the findings should be interpreted in relation to this specific educational context. Differences in instructional delivery and clinical training across institutions may influence the applicability of the results, underscoring the need for replication in diverse academic settings.

## Limitations

This study has several limitations that should be considered when interpreting its findings. First, the use of a single-group quasi-experimental design limits causal inference, as the absence of a control or comparison group makes it difficult to rule out alternative explanations such as maturation, testing effects, or regression to the mean. Second, the sample consisted of volunteer nursing students from a single institution, which may restrict the generalizability of the findings to nursing students in other educational settings with different curricular structures, instructional approaches, or clinical training models. Although nursing programs in Türkiye share nationally defined core requirements, institutional differences may influence students’ time-management behaviors and their responses to intervention.

Another important limitation is the exclusive reliance on self-report measures. While self-report instruments are useful for capturing perceived changes in time management and procrastination, they may be influenced by response bias, including social desirability or increased self-awareness following the intervention, and may not fully reflect actual behavioral change. In addition, the relatively short duration of the intervention and the absence of short-term or long-term follow-up assessments limit conclusions regarding the sustainability of the observed improvements. Without follow-up data, it remains unclear whether reductions in academic procrastination and improvements in time use represent enduring behavioral change or short-term post-intervention effects. Future research would benefit from randomized controlled designs, multi-site sampling, extended follow-up periods, and the inclusion of objective behavioral indicators—such as digital time-use records or task-completion logs—to provide a more comprehensive understanding of the long-term effects of time-management interventions on academic self-regulation.

## Conclusion

This study shows that clear, well-structured, and theory-informed instruction can help nursing students strengthen their time-management behaviors and reduce academic procrastination. Although general procrastination tendencies remained unchanged, the improvements observed in academic behaviors suggest that meaningful change is possible when students receive guidance that supports engagement, clarity, and purposeful practice.

Going forward, nursing programs may benefit from integrating time-management training more intentionally across both classroom and clinical settings, allowing students to apply these skills in real learning environments. Faculty development focused on effective instruction and supportive feedback can further enhance students’ self-regulation. Because deeper procrastination patterns often relate to stress and coping, offering mentoring, digital planning tools, and well-being resources may help sustain long-term behavioral change. By fostering a supportive and responsive educational environment, nursing schools can better equip students for the complex demands of academic work and the realities of professional nursing practice.

## Data Availability

The data supporting the findings of this study are not publicly available due to ethical and privacy considerations but are available from the corresponding author upon reasonable request.
